# Development and pilot testing of the quality of life of parents of children with achondroplasia questionnaire

**DOI:** 10.1186/s41687-026-01127-9

**Published:** 2026-06-19

**Authors:** Adekunle Adedeji, Stefanie Witt, Maria Salomão, Tabea Steinhoff, Florian Innig, Inês Alves, Chiara Provasi, Marco Sessa, Klaus Mohnike, Julia Quitmann

**Affiliations:** 1 Faculty of Social Work and Childhood Education, HAW Hamburg, Hamburg, Germany; 2 Institute of Medical Psychology, University Medical Center Hamburg-Eppendorf, Hamburg, Germany; 3 Institute of Medical Psychology, University Medical Center Hamburg-Eppendorf, Hamburg, Germany; 4 Faculty of Social Work and Childhood Education, HAW Hamburg, Hamburg, Germany; 5 Federal Association for People of Short Stature and their Families (Bundesverband Kleinwüchsige Menschen und ihre Familien e.V.), BKMF, Bremen, Germany; 6 National Association for Skeletal Dysplasias - ANDO Portugal, Evora, Portugal; 7 School of Health and Human Development, University of Évora, Evora, Portugal; 8 Italian Association on Achondroplasia (Associazione per l’Informazione e lo studio dell’acondroplasia) AISAC, Milan, Italy; 9 Children’s Hospital, Otto-von-Guericke University Magdeburg, Magdeburg, Germany; 10 Federal Association for People of Short Stature and their Families (Bundesverband Kleinwüchsige Menschen und ihre Familien e.V.), BKMF, Bremen, Germany

**Keywords:** Achondroplasia, Parents, Quality of life, Caregiver burden, Questionnaire development, Pilot study

## Abstract

**Background:**

Parents of children with achondroplasia face sustained caregiving demands that may affect multiple dimensions of well-being. Despite growing recognition of these challenges, no validated, condition-specific instrument exists to assess the quality of life (QoL) of parents of children with achondroplasia. This study aimed to develop, and pilot test the Quality of Life of Parents of Children with Achondroplasia (QOLA) questionnaire.

**Methods:**

QOLA was developed using a multi-phase mixed-methods design in accordance with established standards for developing self-reported outcome measures for caregivers and parents. Phase 1 comprised semi-structured qualitative interviews with 17 parents of children with achondroplasia to identify relevant QoL domains and language. Interview data were analysed using qualitative content analysis and informed systematic item generation (Phase 2). Conceptual structure was examined through researcher-led card sorting (Phase 3) and two rounds of international card sorting following translation (Phase 4). The resulting 63-item questionnaire across eight domains was pilot-tested in a cross-sectional, multi-country study with embedded cognitive debriefing in Germany, Italy, and Portugal (total *N* = 50).

**Results:**

The final pilot version of QOLA comprised 63 items across eight domains covering healthcare experiences, challenges and support, physical health, mental health, social life and relationships, coping, family and daily life, and worries and future concerns. Item-level missing data were minimal, and no pronounced floor or ceiling effects were observed. Internal consistency was acceptable to good for domains (α = 0.624–0.821) and good for the total scale (α = 0.798). Inter-domain correlations were generally moderate to strong. Cognitive debriefing was highly acceptable and relevant across countries, with some suggestions for further refinement.

**Conclusions:**

QOLA shows strong preliminary evidence of acceptability and internal consistency and addresses a key measurement gap in achondroplasia research. Further large-scale psychometric validation is warranted.

**Supplementary Information:**

The online version contains supplementary material available at 10.1186/s41687-026-01127-9.

## Keypoints

QOLA is a new questionnaire that captures the everyday challenges and well-being of parents of children with achondroplasia. It is understandable, relevant, and shows promising reliability across multiple life domains. Further large-scale validation will help guide support and care for affected families.

**Supplementary Information:**

The online version contains supplementary material available at 10.1186/s41687-026-01127-9.

## Background

Caregiving burden represents a central mechanism through which chronic and rare childhood conditions affect parental well-being. It is increasingly understood as a multidimensional construct encompassing physical strain, emotional exhaustion, time pressure, financial demands, and constraints on social participation. Across paediatric chronic illness contexts, sustained caregiving burden has been consistently associated with reduced health-related quality of life, elevated psychological distress, and cumulative fatigue among parents, particularly when care demands are long-term and unpredictable [1, 2]. Importantly, impaired parental well-being is not only an outcome of caregiving burden, but it also shapes caregiving capacity itself. Higher levels of parental stress and reduced quality of life have been linked to diminished coping resources, difficulties in care coordination, and strain within family systems, with potential downstream effects on family functioning and child outcomes [3]. In conditions characterised by sustained and developmentally evolving care demands, systematic assessment of caregiving burden and its impact on parental quality of life is therefore essential for understanding both parental outcomes and the broader caregiving context.

Achondroplasia is the most common form of skeletal dysplasia and is characterised by disproportionate short stature and may face a range of medical, functional, and psychosocial challenges across the life course [4]. While advances in clinical management have improved health outcomes for individuals with achondroplasia, the condition remains associated with recurrent medical monitoring, specialised care, and ongoing adaptation of the home and social environment. These healthcare and caregiving demands extend beyond the affected individual and substantially shape parents’ experiences, as they may assume a heavier sense of long-term caregiving responsibilities from early childhood onward.

Empirical research has consistently shown that parenting a child with a chronic or rare condition is associated with increased caregiving burden, emotional strain, time pressure, and disruptions to family, occupational, and social functioning [1, 2]. In the context of achondroplasia, parents often navigate complex care pathways involving multiple medical specialties, frequent appointments, and uncertainty regarding long-term functional outcomes [5, 6]. Qualitative studies focusing specifically on parents of children with achondroplasia further demonstrate that caregiving extends far beyond medical management and substantially shapes parental well-being. Parents frequently describe emotional distress following diagnosis, persistent uncertainty regarding their child’s long-term health and independence, and considerable strain related to repeated medical appointments, treatment decisions, and navigating specialised healthcare systems [7–9]. In addition, families report disruptions to work and family routines, concerns about social participation and future autonomy, and the ongoing challenge of balancing protective caregiving with fostering independence and inclusion [8, 9]. Studies focusing on daily treatment management, including vosoritide administration, further highlight the practical and emotional burden associated with sustained caregiving responsibilities [10, 11]. Beyond medical demands, parents also report challenges related to environmental accessibility, social inclusion, stigma, and concerns about their child’s future independence and social participation. Similar patterns have also been described in qualitative studies involving parents of children with short stature more broadly, although these findings are not specific to achondroplasia and should therefore be interpreted as supportive rather than directly transferable evidence [12].

Parental well-being is increasingly recognised as a critical component of family functioning and child outcomes in chronic health contexts. Higher levels of parental stress and reduced quality of life have been linked to poorer psychological adjustment, diminished coping capacity, and strain on family relationships, with potential downstream effects on children’s emotional and social development [3]. Consequently, understanding and systematically assessing parental quality of life is essential for both clinical practice and research, particularly in conditions characterised by lifelong and fluctuating care needs such as achondroplasia.

Despite this recognition, the assessment of parental quality of life in achondroplasia has largely relied on generic quality-of-life instruments or broad caregiver burden measures. While such instruments facilitate comparisons across populations, they often lack sensitivity to condition-specific experiences and may fail to capture domains that are particularly salient to parents of children with achondroplasia, including navigation of specialised healthcare systems, environmental adaptations, social visibility and stigma, and the ongoing balance between protective caregiving and fostering autonomy [13, 14]. Previous empirical studies, including qualitative and observational research, indicate that these condition-specific challenges are central to parental experiences but remain insufficiently represented in existing measurement approaches [5, 15]. Although some caregiving challenges may overlap with those reported in other rare or chronic childhood conditions, achondroplasia presents a distinct combination of lifelong medical monitoring, specialised multidisciplinary care, visible physical difference, and ongoing environmental adaptation that uniquely shapes parental quality of life. Parents must often balance protective caregiving with supporting autonomy and social participation in ways that are closely linked to the specific functional and social realities of achondroplasia. Including broader groups of short stature conditions or skeletal dysplasias could reduce content validity, as important experiences related to visibility, healthcare navigation, and future independence may differ substantially across diagnoses. For this reason, the present study focused specifically on parents of children with achondroplasia.

Despite this recognition, the assessment of parental quality of life in achondroplasia has largely relied on generic quality-of-life instruments or broad caregiver burden measures. While such instruments facilitate comparisons across populations, they often lack sensitivity to condition-specific experiences and may fail to capture domains that are particularly salient to parents of children with achondroplasia, including navigation of specialised healthcare systems, environmental adaptations, social visibility and stigma, and the ongoing balance between protective caregiving and fostering autonomy [13, 14]. Previous empirical studies, including qualitative and observational research, indicate that these condition-specific challenges are central to parental experiences but remain insufficiently represented in existing measurement approaches [5, 15]. Although some caregiving challenges overlap with other rare childhood conditions, achondroplasia involves a distinct combination of lifelong medical monitoring, specialised care, visible physical difference, and environmental adaptation that uniquely shapes parental quality of life. Including broader short stature conditions or other skeletal dysplasias could reduce content validity and limit sensitivity to achondroplasia-specific experiences that uniquely shapes parental quality of life [4, 6, 7].

Disease-specific patient- and proxy-reported outcome measures have been shown to provide greater content validity and responsiveness by reflecting the language, priorities, and contextual realities of the target population [16, 17]. Current methodological standards therefore emphasise the importance of grounding questionnaire development in qualitative research with the affected population, followed by systematic item generation, refinement, and pilot testing [14, 18]. To date, however, no validated questionnaire specifically designed to assess the self-reported quality of life of parents of children with achondroplasia is available.

The absence of a condition-specific, parent self-reported quality-of-life instrument limits the ability to comprehensively assess parental well-being, identify unmet support needs, and evaluate the broader impact of clinical and psychosocial interventions. A condition-specific measure co-developed with parents, is therefore needed to capture the multidimensional nature of parental quality of life when raising a child with achondroplasia, encompassing healthcare experiences, physical and mental health, family functioning, social participation, coping, and future-related concerns. The development of such an instrument represents a necessary step toward improving outcome assessment in both clinical care and research involving families affected by achondroplasia.

## Methods

### Study design and overall development process

**Fig. 1:**
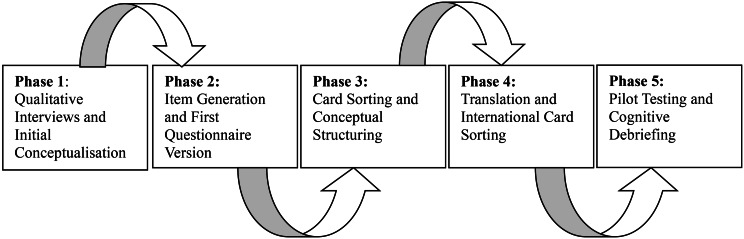
Project flow diagram

The QOLA questionnaire was developed using a multi-phase, mixed qualitative and quantitative approach in accordance with established recommendations for developing patient-reported outcome measures (See Fig. 1 below) [14]. The development process combined qualitative interviews, structured qualitative content analysis, iterative item generation, card sorting procedures, cross-cultural evaluation, and pilot testing with cognitive debriefing. The overall aim was to ensure strong content validity, conceptual clarity, and cross-cultural applicability of the instrument. Ethical approval was obtained from the Local Psychological Ethics Committee at the University Medical Centre Hamburg-Eppendorf (UKE).

### Phase 1: Qualitative interviews with parents and initial conceptualisation

#### Design and aim

Phase 1 aimed to establish the empirical and conceptual foundation of the QOLA questionnaire through qualitative exploration of parental experiences of caring for a child with achondroplasia. In-depth, semi-structured interviews were conducted to identify domains, themes, and language relevant to parental quality of life, in accordance with content validity requirements for developing patient-reported outcome measures [14, 16].

#### Participants and recruitment

Parents of children with a confirmed diagnosis of achondroplasia were eligible to participate if the identified has the primary caregiver and sufficiently fluent in German to engage in an in-depth interview. Participants were recruited through clinical networks and patient organisations collaborating in the QOLA project. A purposive sampling strategy was employed to ensure heterogeneity in children’s ages, as recommended for qualitative instrument development [19]. Recruitment continued until thematic saturation was achieved [20]. In total, 17 parents participated in Phase 1.

#### Interview guide

Data collection was guided by a semi-structured interview guide specifically developed for the QOLA project. The guide was designed to comprehensively cover parental experiences across the illness trajectory while allowing flexibility to explore topics relevant to each individual, consistent with best practices in qualitative health research [21]. Core thematic areas included: experiences prior and during the diagnosis, the diagnostic process and communication of the diagnosis, emotional responses and evolving perspectives, informational and support needs, interactions with healthcare providers, changes in daily life and family dynamics (including partner relationships, siblings, work, and leisure), psychosocial burden, coping resources, and future-related worries and expectations. The guide also explicitly encouraged reflection on potentially positive experiences, sources of meaning, and resilience building.

The interview guide was developed by an interdisciplinary research team, drawing on clinical expertise, a review of relevant literature, and preliminary input from parents and patient representatives, in accordance with COSMIN recommendations for content validity [18]. Open-ended questions were supplemented by optional probes to facilitate depth and clarification. The final interview guide is provided as Supplementary Material, with additional parent-generated thematic suggestions documented separately.

#### Interview procedure

Participants received written study information prior to participation. At the start of each interview, the interviewer introduced themselves, explained the study aims, confirmed informed consent, and obtained permission for audio recording. Participants were reminded that participation was voluntary and that they could withdraw at any time without consequences.

Interviews were conducted in German by two researchers (TS and AA), both with experience in psychosocial research, patient-reported outcomes, and qualitative methods in rare disease contexts. Neither interviewer was involved in the clinical care of participants, and no prior therapeutic relationship existed between interviewers and participants. Their roles were explained at the beginning of each interview to minimise role ambiguity and reduce potential response bias. Interviews were conducted either via telephone or video call, depending on participant preference. Interviews were conducted in a quiet, private setting and followed the interview guide, allowing participants to elaborate freely on topics of personal relevance. Interview duration ranged from approximately 40 to 60 min, consistent with recommendations for in-depth qualitative interviews [22]. All interviews were audio-recorded, transcribed verbatim, and anonymised. Audio files were stored securely and deleted after transcription in accordance with data protection regulations. Field notes were recorded after each interview to capture contextual information and analytic reflections.

#### Qualitative data analysis

Interview transcripts were analysed using qualitative content analysis following the approach described by Mayring (2014). Analysis combined deductive and inductive procedures. Deductive main categories were informed by the interview guide and the overarching concept of parental quality of life, while inductive subcategories were developed directly from the interview material.

Coding was conducted using MAXQDA (VERBI Software, Berlin, Germany). An initial coding framework was developed from a subset of transcripts and iteratively refined throughout the analysis. Coding decisions and category definitions were regularly discussed within the research team to enhance analytic rigour, reflexivity, and conceptual coherence [23]. The final qualitative structure comprised 14 main categories and 28 subcategories, representing core dimensions of parental quality of life in the context of achondroplasia and serving as the basis for subsequent item generation.

### Phase 2: Item generation and first questionnaire version

Candidate questionnaire items were developed from the qualitative categories identified in Phase 1. Verbatim quotations and thematic summaries were systematically translated into item formulations by an interdisciplinary research team, following established guidelines for patient-reported outcome measure (PROM) development [16, 18]. Item wording was anchored in parents’ original expressions wherever possible and refined to ensure clarity, conceptual precision, and suitability for self-report assessment, with a focus on experiences and perceptions rather than clinical facts [17].

The initial item pool underwent internal review to identify unclear wording, overlapping content, and redundancies. Items were refined to improve readability and conceptual distinctiveness prior to further structuring. This process produced the first German-language questionnaire version, reflecting the full conceptual scope of parental quality of life identified in the qualitative analysis.

### Phase 3: Card sorting and conceptual structuring

To examine conceptual coherence and domain structure, a researcher-led card sorting procedure was conducted using the German-language item pool. Individual items were presented as cards and grouped into conceptually coherent domains based on thematic similarity, following established approaches to questionnaire structuring [24].

The card sorting was conducted in an open, exploratory format by three researchers, who independently assigned items to domains and documented uncertainties, overlaps, and ambiguities in item wording or domain allocation. Qualitative feedback was recorded during the procedure. The resulting groupings and annotations were synthesised by the research team to inform refinement of domain boundaries and item formulations, ensuring conceptual clarity and theoretical coherence prior to cross-cultural evaluation [18].

### Phase 4: Translation and international card sorting

Following conceptual refinement, the German questionnaire was translated into English to facilitate international collaboration and cross-cultural evaluation. Translation was conducted using a forward translation approach by bilingual researchers with expertise in health measurement and familiarity with the subject matter, in accordance with recommended practices for early-stage instrument [25].

Using the English version, two successive international card sorting exercises were conducted to assess conceptual coherence, domain structure, and cross-cultural interpretability. Participants grouped items into conceptually meaningful domains and provided qualitative feedback on clarity, relevance, and cultural appropriateness. Feedback from each round was qualitatively synthesised and used to guide iterative refinement of item wording, domain structure, and item inclusion. This process aimed to ensure cross-cultural conceptual equivalence and content validity prior to pilot testing, consistent with COSMIN recommendations [18].

### Phase 5: Pilot testing and cognitive debriefing

#### Pilot version and study design

The pilot phase aimed to evaluate the clarity, relevance, and acceptability of the refined QOLA questionnaire and to identify remaining issues prior to large-scale psychometric validation. The pilot version consisted of 63 items organised into 8 domains, reflecting the consolidated conceptual structure derived from the international card-sorting process (Phase 4). A multi-country pilot study with embedded cognitive debriefing interviews was conducted in Germany, Italy, and Portugal using a cross-sectional design.

#### Participants and recruitment

In each participating country, 16–17 parents of children with achondroplasia were recruited, resulting in a total pilot sample of *N* = 50. Eligible participants were primary caregivers of a child with a confirmed diagnosis of achondroplasia and sufficient fluency in the study language to complete the questionnaire and participate in cognitive debriefing. Recruitment was facilitated through established patient organisations in each country. In Germany, participants were recruited through the Bundesverband Kleinwüchsige Menschen und ihre Familien e.V. (BKMF). In Italy, recruitment was supported by AISAC APS (Associazione Italiana Sostegno Achondroplasia), and in Portugal by ANDO Portugal (Associação Nacional de Displasias Ósseas). These organisations disseminated study information via established communication channels, including mailing lists, newsletters, and online platforms.

#### Pilot procedure

Participants completed the pilot questionnaire in their respective language versions (German, Italian, or Portuguese). Upon completion, participants took part in cognitive debriefing interviews conducted by trained researchers. Cognitive debriefing interviews were conducted individually, either by telephone or video call, and followed a semi-structured format. For the cognitive debriefing, each questionnaire item was systematically evaluated by participants for clarity, relevance, and appropriateness, and participants were asked whether rewording was needed. Participants were encouraged to explain their interpretations of items, highlight ambiguities, and describe any difficulties encountered during questionnaire completion.

In addition to item-level feedback, participants were asked to evaluate the questionnaire as a whole using the same criteria (clarity, relevance, appropriateness, and completeness). This included reflections on the overall length of the questionnaire, perceived redundancy, and whether important aspects of their experience as parents of a child with achondroplasia were missing.

#### Data analysis

Pilot questionnaire data were analysed descriptively to examine response distributions, patterns of missing data, and potential floor or ceiling effects at the item and domain levels, in accordance with recommendations for pilot testing of patient-reported outcome measures [13]. These analyses were used to identify items with limited variability or systematic non-response indicative of potential comprehension or relevance issues. Preliminary internal consistency was assessed using Cronbach’s alpha coefficients for each domain in the pooled pilot sample. Item–domain correlations were examined to evaluate alignment between individual items and their intended domains [13, 26]. Given the exploratory nature of the pilot study, reliability findings were interpreted descriptively and used to inform item refinement rather than to establish definitive psychometric properties. In addition to internal consistency analyses, item–domain relationships were examined to assess the contribution of individual items to their intended domains and to identify potential redundancy. Exploratory factor-analytic evidence was used to evaluate domain structure, item loadings, and cross-loadings, supporting decisions regarding item retention, removal, and refinement. These statistical findings were interpreted alongside cognitive debriefing feedback, item-level response distributions, and qualitative review to ensure that psychometric refinement did not compromise content validity, in line with COSMIN recommendations [9, 13, 20].

Cross-country comparability of domain scores across Germany, Italy, and Portugal was explored using descriptive statistics and inferential analyses (t-tests or analyses of variance, as appropriate). In addition, random-intercept mixed-effects models were fitted for each domain, with country specified as a grouping factor, to estimate variance partition coefficients reflecting the proportion of variance attributable to between-country differences. These ICC-like estimates were interpreted descriptively as indicators of country-level clustering rather than as classical intraclass correlation coefficients, which require repeated measurements of the same individuals across conditions [27]. Sociodemographic and socioeconomic variables were examined to assess whether observed cross-country differences were attributable to sample composition.

Cognitive debriefing data were analysed using a mixed descriptive approach consistent with best practices for cognitive interviewing [28, 29]. Quantitative ratings of item clarity, relevance, appropriateness, and need for rewording were summarised descriptively, alongside overall questionnaire-level evaluations of acceptability and completeness. Open-ended feedback was analysed using structured content analysis [23]. Items showing consistently low ratings or recurring qualitative concerns across participants or countries were flagged for revision or removal in line with COSMIN recommendations for content validity assessment [18].

## Results

### Phase 1: Qualitative interviews (concept elicitation)

Qualitative interviews were conducted with parents of children diagnosed with achondroplasia, including three fathers, with the remaining participants being mothers. The mean age of the children was 8.9 years (SD = 4.2; range 3–17 years).

Interviews revealed a multidimensional pattern of parental experiences spanning emotional, physical, organisational, relational, and future-oriented domains. Parents described cumulative caregiving demands that affected their well-being, including emotional exhaustion, physical strain, and ongoing adaptation of family, work, and daily routines. Daily life was characterised by ongoing organisational efforts, such as environmental adaptations, coordination of medical and therapeutic appointments, and negotiation of inclusion in childcare, school, and community settings. The diagnostic period was recalled as emotionally intense and marked by uncertainty, while interactions with healthcare systems and administrative authorities constituted persistent sources of strain. Alongside these challenges, parents reported active coping strategies, positive reappraisals, and the importance of peer exchange and patient organisations. Future-oriented concerns regarding children’s health, autonomy, social participation, and long-term life prospects were prominent. Overall, the qualitative analysis yielded 28 empirically derived codes that informed subsequent item generation and conceptual structuring.

### Phase 2: Item generation (initial pool)

Building on the qualitative findings from Phase 1, the 28 empirically derived codes served as the foundation for systematic item generation. Verbatim quotations, paraphrased statements, and thematic summaries were translated into candidate questionnaire items that reflected parental experiences, priorities, and language as closely as possible. Item formulation focused on capturing subjective perceptions and experiences rather than objective clinical characteristics.

During this process, all qualitatively identified content areas were adequately represented, including emotional burden, everyday caregiving demands, family dynamics, healthcare experiences, social participation, coping processes, and future-related concerns, among others (see ST1 in Supplementary Materials for a full list). Items were worded to be applicable across different child ages and caregiving contexts and to allow for variability in individual experiences. This iterative process resulted in the first German-language version of the QOLA questionnaire, comprising 73 items organised into 14 preliminary categories. This initial questionnaire version served as the basis for subsequent conceptual structuring and refinement through card-sorting procedures in Phase 3.

### Phase 3: National card sorting (within-language structuring)

Researcher-led card sorting was used to examine the conceptual coherence of the initial item pool. Overall, the results supported the qualitative category structure derived in Phase 2. Most items were consistently assigned to the same thematic clusters, indicating strong conceptual clarity.

At the same time, card sorting highlighted areas of overlap between categories and a small number of items with ambiguous domain placement. These insights informed minor refinements to category boundaries and item wording. Importantly, this phase confirmed the 14-category structure, providing a stable conceptual foundation for cross-cultural evaluation.

### Phase 4: International card sorting (cross-cultural consolidation)

Following Phase 3, the 73-item German questionnaire was translated into English to facilitate international collaboration and cross-cultural evaluation. Forward translation was followed by independent back-translation into German, Italian, and Portuguese, enabling systematic comparison of the source and target versions. Discrepancies were discussed within the multilingual research team and resolved by consensus, resulting in linguistically aligned versions across languages. International card sorting using the English version revealed substantial conceptual overlap among several categories, supporting consolidation into broader domains. Based on these findings, items were regrouped and redundancies reduced, yielding a revised structure comprising 66 items organised into 8 overarching categories.

A second international card sorting exercise was conducted using the revised item set. This round further refined domain boundaries and item clarity across cultural contexts. Additional items were reworded or removed based on conceptual fit and cross-language interpretability, resulting in a consolidated version comprising 63 items across the same 8 domains: healthcare experiences, challenges and support, physical health, mental health, social life and relationships, coping, family and daily life, and worries and future concerns. The consolidated 63-item questionnaire was subsequently forward- and back-translated into German, Italian, and Portuguese to ensure linguistic equivalence and cross-cultural applicability of all final items. This multilingual version formed the basis for pilot testing and cognitive debriefing.

### Phase 5: Pilot testing and cognitive debriefing (multinational)

#### Sample characteristics

A total of 51 parents were recruited across Germany, Italy, and Portugal; 50 provided complete pilot data and were included in analyses (Germany *n* = 16; Italy *n* = 17; Portugal *n* = 17). Item-level missingness was minimal, and response distributions showed adequate variability without pronounced floor/ceiling effects. Domain scores were approximately continuous and suitable for preliminary reliability and correlational analyses.

#### Internal consistency reliability

In the pooled sample (*N* = 50), internal consistency was was generally acceptable to good across domains and for the total scale (Table 1). Reliability was consistently acceptable in the German and Portuguese subsamples. In the Italian subsample, domain-level estimates were more heterogeneous, with lower values in some domains and acceptable-to-good values in others, while the total score remained acceptable. Given the small pilot sample size, particularly within language-specific subsamples, Cronbach’s alpha estimates should be interpreted cautiously, as reliability coefficients may be inflated by restricted variance and item homogeneity at this stage of development.

**Table 1:** Internal consistency (Cronbach’s α) of pilot QOLA domains (63 items)

Domain	No. of items	Pooled sample (*N* = 50)	German (*n* = 16)	Portuguese (*n* = 17)	Italian (*n* = 17)
Healthcare	9	0.671	0.766	0.833	0.524
Accommodations	7	0.650	0.680	0.641	0.665
Physical Health	6	0.639	0.776	0.608	0.474
Mental Health	9	0.706	0.610	0.809	0.709
Social Interactions	8	0.640	0.800	0.409	0.555
Support	6	0.624	0.508	0.547	0.483
Everyday Life	10	0.821	0.865	0.801	0.748
Future Worries	8	0.786	0.656	0.790	0.778
Total scale	63	0.798	0.767	0.796	0.852

Note. Cronbach’s alpha coefficients are reported for the pooled pilot sample and separately by language version. Results should be interpreted descriptively, given the pilot nature of the study and the small sample sizes in the language-specific analyses

#### Inter-domain associations

In the pooled sample, inter-domain correlations were generally moderate to strong, with correlations ranging from *r* = − .724 to *r* = .645, and with most reaching statistical significance (Table 2). The strongest associations were observed between challenges and support and mental health, and between support/coping and future worries, consistent with the interrelated nature of caregiving stressors. Germany showed uniformly strong correlations, while Portugal exhibited moderate-to-strong associations. Italy showed more heterogeneous patterns, with several non-significant correlations among domains with lower reliability, and stronger associations among mental health, future worries, everyday life, and challenges and support.

**Table 2:** Summary of inter-domain associations across samples, including statistical significance

Sample	*N*	Range of Pearson correlations (*r*)	Statistical significance	Notable strong associations
Pooled sample	50	− 0.724 – 0.645	Most *p* < .001; a few non-significant (e.g., HE–WF, Co–WF)	Challenges–Mental Health (*r* = .645); Support–Future Worries (*r* = .724)
Germany	16	− 0.827 – 0.815	Most *p* < .05; some borderline non-significant	Social Interactions–Support (*r* = − .827); Social Interactions–Everyday Life (*r* = .815)
Portugal	17	. − 0.679 – 0.738	Most *p* ≤ .05	Challenges–Mental Health (*r* = .738); Mental Health–Social Interactions (*r* = .733)
Italy	17	− 0.570 – 0.811	Several non-significant correlations	Mental Health–Future Worries (*r* = .811); Mental Health–Everyday Life (*r* = .757)

Note. Pearson correlation coefficients are reported for domain-level scores. Full correlation matrices are available in the Supplementary Materials (ST 4 to 8)

#### Cross-country comparability and country-level clustering

To examine the extent of country-level clustering, random-intercept mixed-effects models were fitted for each domain with country specified as the grouping factor.

For the Everyday Life (FD) domain, the estimated between-country variance was 0.106 (SE = 0.133), and the residual variance was 0.436 (SE = 0.090), yielding a variance partition coefficient (ICC-like estimate) of 0.195. For the Future Worries (WF) domain, the estimated between-country variance was 0.205 (SE = 0.230), with a residual variance of 0.415 (SE = 0.086), corresponding to an ICC-like estimate of 0.331. These results indicate that, although some country-level clustering was present, the majority of variance in both domains was attributable to within-country differences rather than between-country effects.

### Cognitive debriefing results

Cognitive debriefing indicated high acceptability and perceived relevance across countries. Most respondents rated the questionnaire as easy to complete and judged the content as accurate and relevant. Perceived completeness showed greater variability, with a higher proportion of respondents in Germany and Italy indicating that some relevant aspects might still be missing (Table 3).

**Table 3:** Cognitive debriefing results: response distributions for questionnaire acceptability, relevance, and completeness (pooled sample and by country)

Domain	Response category	Pooled (*N* = 50)	Germany (*n* = 16)	Portugal (*n* = 17)	Italy (*n* = 17)
The questionnaire is easy to complete	Very easy	11 (22.0%)	1 (6.3%)	5 (29.4%)	5 (29.4%)
	Easy	25 (50.0%)	6 (37.5%)	11 (64.7%)	8 (47.1%)
	Neither difficult nor easy	12 (24.0%)	7 (43.8%)	1 (5.9%)	4 (23.5%)
	Difficult	1 (2.0%)	1 (6.3%)	–	–
	Very difficult	1 (2.0%)	1 (6.3%)	–	–
The questions are accurate	Not at all	1 (2.0%)	–	–	1 (5.9%)
	A little	2 (4.0%)	1 (6.3%)	1 (5.9%)	–
	To some extent	6 (12.0%)	4 (25.0%)	–	2 (11.8%)
	Very much	35 (70.0%)	10 (62.5%)	13 (76.5%)	12 (70.6%)
	Completely	6 (12.0%)	1 (6.3%)	3 (17.6%)	2 (11.8%)
The questionnaire relevant	Not at all	1 (2.0%)	–	–	1 (5.9%)
	To some extent	4 (8.0%)	3 (18.8%)	–	1 (5.9%)
	Very much	34 (68.0%)	11 (68.8%)	12 (70.6%)	11 (64.7%)
	Completely	11 (22.0%)	2 (12.5%)	5 (29.4%)	4 (23.5%)
The questionnaire is incomplete	Totally disagree	7 (14.0%)	2 (12.5%)	1 (5.9%)	4 (23.5%)
	Disagree	22 (44.0%)	4 (25.0%)	11 (64.7%)	7 (41.2%)
	Neither agree nor disagree	6 (12.0%)	3 (18.8%)	3 (17.6%)	–
	Agree	13 (26.0%)	6 (37.5%)	2 (11.8%)	5 (29.4%)
	Totally agree	2 (4.0%)	1 (6.3%)	–	1 (5.9%)

### Post-debriefing refinement and finalisation of the field-test version

Pilot findings were integrated into a structured refinement process combining (i) cognitive debriefing feedback, (ii) item-level distributional checks, (iii) domain-level reliability patterns, and (iv) exploratory factor-analytic evidence. Item reduction prioritised factor-analytic criteria (low/ambiguous loadings; improvement of domain structure and internal consistency after item removal) while safeguarding content validity through qualitative review of parent feedback and domain coverage. This resulted in a reduction from 63 to 43 items, retaining the same eight-domain framework (healthcare experiences; challenges and support needs; physical health; mental health; social interactions; coping; daily functioning; worries and future concerns). Item refinement process is provided in ST2, and reliability values for the refined version (43-item) in ST3, both in Supplementary Materials.

Given weaker reliability patterns and more heterogeneous associations in Italy, additional steps were undertaken for the Italian version, including focused re-checking of domain allocation (card sorting), targeted revision of item wording to improve conceptual equivalence, and stakeholder-based cognitive review with representatives of the Italian patient organisation. This process supported improved clarity and informed minor final wording adjustments. The harmonised 43-item field-test version is provided in ST4 in Supplementary Materials.

## Discussion

This study reports the development, cross-cultural refinement, and pilot evaluation of the Quality of Life of Parents of Children with Achondroplasia (QOLA), a condition-specific parent self-reported outcome measure designed to capture the multidimensional impact of parenting a child with achondroplasia. The instrument responds to a recurring limitation in paediatric rare-disease research: parental outcomes are often assessed using generic caregiver burden or family-impact measures that enable comparability but may not adequately reflect condition-specific stressors, healthcare-system interactions, and the distinctive social visibility and stigma that can shape daily life in achondroplasia [1, 13, 30, 31]. By grounding item content in parent narratives and iteratively testing conceptual structure across countries, QOLA advances the precision of parental outcome measurement in this population.

Importantly, domains such as healthcare experiences and support needs were intentionally included because, in caregiving contexts, quality of life is shaped not only by internal psychological well-being but also by the broader systems within which caregiving occurs. Repeated navigation of specialised healthcare services, administrative processes, access to support, and institutional trust function as chronic sources of stress and directly influence emotional burden, coping capacity, and daily functioning. In rare conditions such as achondroplasia, these external structures are closely intertwined with parental well-being rather than representing separate contextual variables. Consistent with caregiving burden models and family systems perspectives, QOLA therefore conceptualises healthcare experiences and support needs as integral components of parental quality of life rather than merely background service factors [1–3].

### Interpreting the content domains in light of caregiving theory and achondroplasia-specific demands

The qualitative foundation of QOLA aligns strongly with contemporary models conceptualising caregiving as a dynamic configuration of stressors (e.g., time demands, uncertainty, physical strain) and resources (e.g., coping, social support, meaning making) that jointly shape parental well-being over time [2, 3]. Parents described burden-related impacts (emotional exhaustion, physical strain, organisational load) alongside adaptive processes (positive reappraisal, reliance on peer exchange and patient organisations), underscoring the importance of representing both vulnerability and resilience-related content rather than treating caregiving solely as unidimensional strain [1, 3]. In achondroplasia specifically, these experiences are plausibly amplified or patterned by (i) sustained interactions with specialised healthcare and administrative systems, (ii) chronic anticipatory concerns about functional development and autonomy, and (iii) high social visibility that can increase the frequency of public interactions, disclosure, and stigma management [4–6, 15].

### Evidence supporting the proposed structure and feasibility of the field-test version

Pilot findings provide initial support for the coherence and feasibility of the QOLA framework. Minimal missing data and adequate response variability suggest that parents across countries could engage with the items and response format, consistent with best practices in PROM development that emphasise comprehensibility and acceptability as prerequisites for robust measurement [18, 28, 29]. Domain-level internal consistency was generally acceptable to good in the pooled sample (α = 0.624–0.821), and the total scale showed good reliability (α = 0.798), providing early evidence that the domains function as internally coherent sets of indicators. Reliability was relatively consistent in the German and Portuguese subsamples, while the Italian subsample showed more heterogeneous domain-level reliability, with several domains falling below conventional thresholds, though the total score remained acceptable. Importantly, reliability evidence alone is not sufficient to claim structural validity; however, in early-stage instrument development it offers a useful screening signal when interpreted alongside conceptual clarity and qualitative feedback [13, 26].

Inter-domain correlations in the pooled sample were generally moderate to strong (*r* = − .724 to 0.645), with most reaching statistical significance, reflecting theoretically expected interdependence among caregiving burden dimensions (e.g., self-reflection, support, daily functioning, and psychosocial strain) described in caregiving models [2]. At the same time, the pattern of correlations does not compel a single undifferentiated construct interpretation; instead, it is consistent with a related-but-distinct domain architecture, which is typical of QoL frameworks and family impact measures where domains represent partially overlapping facets of functioning [13, 31]. The strong coupling between challenges and support and mental health is also plausible in rare disease contexts, where repeated medical decision-making, bureaucratic interactions, and uncertainty can elevate psychological strain and shape perceptions of care [1, 3], and may be particularly salient in achondroplasia due to the multi-specialty care pathways and long-term monitoring described in the clinical literature [4, 6].

### Cross-cultural variability and the Italian pilot pattern: measurement vs. context

The more heterogeneous reliability and correlation patterns in Italy highlight a key issue in multinational PROM development: early versions of measures often display language- and context-sensitive functioning before full linguistic harmonisation and formal invariance testing [18, 25]. Such variability can reflect multiple mechanisms, including (i) translation nuances that shift item meaning, (ii) differential interpretation shaped by healthcare-system organisation or social policy, and (iii) restricted variance in small samples that destabilises reliability coefficients and attenuates correlations. The study’s response to the Italian pattern additional card sorting, targeted linguistic correction, and stakeholder-based cognitive review represents a methodological strength because it treats cross-cultural discrepancies as actionable evidence requiring refinement rather than as noise to be explained post hoc.

### Rationale and implications of reducing to a 43-item field-test version

A major output of the pilot phase is the 43-item field-test version, produced through an explicit integration of qualitative feedback and psychometric indicators. Item reduction was guided primarily by factor-analytic evidence (poor/ambiguous loadings and improved domain structure after item removal) while preserving domain coverage to maintain content validity, a core COSMIN requirement [18]. This is particularly important in QoL measures, where overly aggressive reduction risks narrowing constructs to the most statistically “clean” items at the expense of representing parental experiences [13, 16, 17]. The resulting reduction substantially improves feasibility and respondent burden, an important consideration for parents managing high time pressure and care demands, while retaining the multidimensional framework needed for clinically meaningful profiling and intervention evaluation.

From an applied standpoint, a condition-specific measure such as QOLA can complement generic family-impact tools (e.g., PedsQL Family Impact Module) by increasing sensitivity to achondroplasia-relevant domains (e.g., specialised care navigation, environmental adaptations, stigma-related social experiences) and enabling more targeted needs assessment in clinical and psychosocial care pathways [15, 18, 31]. In research settings, QOLA may support the evaluation of family-centred interventions and policy changes, in which parental functioning is both an outcome and a potential mediator of child participation and adjustment [3].

## Limitations

Several limitations should be considered. First, the pilot sample was small, particularly within language groups, limiting the stability of reliability estimates and the interpretability of cross-country patterns [13]. Second, the pilot design was cross-sectional, precluding examination of test–retest reliability, responsiveness, and longitudinal validity. Third, although cross-cultural refinement was extensive, formal structural validation (e.g., confirmatory factor analysis), measurement invariance testing across countries, and full linguistic validation procedures were beyond the scope of this phase [18, 25]. Fourth, the high inter-domain correlations in some subsamples suggest that future work should explicitly test higher-order structure and evaluate whether a total score, domain scores, or both are optimally interpretable for clinical and research use.

## Conclusions

QOLA is the first condition-specific parent self-reported measure designed to assess quality of life among parents of children with achondroplasia. The multi-phase development process produced an eight-domain framework with strong preliminary feasibility and internal consistency evidence in a multinational pilot. A transparent post-debriefing refinement process yielded a harmonised 43-item field-test version, including targeted optimisation of the Italian version based on cross-cultural evidence and stakeholder input. The next step is large-scale psychometric validation, including confirmatory testing of the domain structure, measurement invariance analyses across countries, test–retest reliability, and responsiveness assessment. Once validated, QOLA has the potential to strengthen family-centred outcome assessment and support more targeted clinical and psychosocial support for families affected by achondroplasia.

## Supplementary Information

Below is the link to the electronic supplementary material.

Supplementary Material 1

## Data Availability

The datasets generated and/or analysed during the current study are available from the corresponding author upon reasonable request.

## Declarations

### Use of artificial intelligence (AI)

including DeepL, Grammarly, and ChatGPT, were utilised to refine sentence structure and enhance language clarity.

## References

[1] Cousino MK, Hazen RA (2013) Parenting Stress Among Caregivers of Children With Chronic Illness: A Systematic Review. J Pediatr Psychol 38(8):809–828. 10.1093/jpepsy/jst049 23843630 10.1093/jpepsy/jst049

[2] Raina P, O’Donnell M, Rosenbaum P, Brehaut J, Walter SD, Russell D et al (2005) The Health and Well-Being of Caregivers of Children With Cerebral Palsy. Pediatrics 115(6):e626–e636. 10.1542/peds.2004-1689 15930188 10.1542/peds.2004-1689

[3] Pinquart M (2018) Parenting stress in caregivers of children with chronic physical condition—A meta-analysis. Stress Health 34(2):197–207. 10.1002/smi.2780 28834111 10.1002/smi.2780

[4] Pauli RM (2019) Achondroplasia: a comprehensive clinical review. Orphanet J Rare Dis 14(1):1. 10.1186/s13023-018-0972-6 30606190 10.1186/s13023-018-0972-6 PMC6318916

[5] Ireland PJ, Donaghey S, McGILL J, Zankl A, Ware RS, Pacey V et al (2012) Development in children with achondroplasia: a prospective clinical cohort study. Develop Med Child Neuro 54(6):532–537. 10.1111/j.1469-8749.2012.04234.x 10.1111/j.1469-8749.2012.04234.x 22409389

[6] Witt S, Kolb B, Bloemeke J, Mohnike K, Bullinger M, Quitmann J (2019) Quality of life of children with achondroplasia and their parents - a German cross-sectional study. Orphanet J Rare Dis 14(1):194. 10.1186/s13023-019-1171-9 31399110 10.1186/s13023-019-1171-9 PMC6688231

[7] Pfeiffer KM, Brod M, Smith A, Viuff D, Ota S, Charlton RW (2021) A qualitative study of the impacts of having an infant or young child with achondroplasia on parent well-being. Orphanet J Rare Dis 16(1):351. 10.1186/s13023-021-01978-z 34362417 10.1186/s13023-021-01978-z PMC8344208

[8] Pfeiffer KM, Brod M, Smith A, Gianettoni J, Viuff D, Ota S et al (2021) Assessing the impacts of having a child with achondroplasia on parent well-being. Qual Life Res 30(1):203–215. 10.1007/s11136-020-02594-3 PubMed PMID: 32803627; PubMed Central PMCID: PMC7847864 32803627 10.1007/s11136-020-02594-3 PMC7847864

[9] Hoover-Fong JE, Savarirayan R, Alves I, Crews C, Haider A, Iruretagoyena SN et al (2026) Qualitative Research in Children and Parents of Children with Achondroplasia to Evaluate the Content Validity of Multiple Clinical Outcome Assessments. Adv Ther 43(1):356–376. 10.1007/s12325-025-03425-y 41313545 10.1007/s12325-025-03425-y PMC12858474

[10] NiMhurchadha S, Butler K, Argent R, Palm K, Baujat G, Cormier-Daire V et al (2023) Parents’ Experience of Administering Vosoritide: A Daily Injectable for Children with Achondroplasia. Adv Ther 40(5):2457–2470. 10.1007/s12325-023-02496-z 37017912 10.1007/s12325-023-02496-z PMC10129947

[11] Semler O, Cormier-Daire V, Lausch E, Bober MB, Carroll R, Sousa SB et al (2024) Vosoritide Therapy in Children with Achondroplasia: Early Experience and Practical Considerations for Clinical Practice. Adv Ther 41(1):198–214. 10.1007/s12325-023-02705-9 PubMed PMID: 37882884; PubMed Central PMCID: PMC10796712 37882884 10.1007/s12325-023-02705-9 PMC10796712

[12] Lackner L, Quitmann JH, Witt S (2023) Caregiving burden and special needs of parents in the care of their short-statured children – a qualitative approach. Front Endocrinol (Lausanne) 14:1093983. 10.3389/fendo.2023.1093983 PubMed PMID: 37008922; PubMed Central PMCID: PMC10064859 37008922 10.3389/fendo.2023.1093983 PMC10064859

[13] De Vet HCW, Terwee CB, Mokkink LB, Knol DL (2011) Measurement in medicine: a practical guide [Internet]. 1st ed. Cambridge University Press. [cited 2026 Feb 5]. Available from: https://doi.org/10.1017/CBO9780511996214https://www.cambridge.org/core/product/identifier/9780511996214/type/book https://doi.org/10.1017/CBO9780511996214

[14] Mokkink LB, Terwee CB, Patrick DL, Alonso J, Stratford PW, Knol DL et al (2010) The COSMIN checklist for assessing the methodological quality of studies on measurement properties of health status measurement instruments: an international Delphi study. Qual Life Res 19(4):539–549. 10.1007/s11136-010-9606-8 20169472 10.1007/s11136-010-9606-8 PMC2852520

[15] Adedeji A, Witt S, Innig F, Alves I, Provasi C, Sessa M et al (2025) Coping and quality of life of parents of children with achondroplasia—a narrative review. Front Med 12:1500389. 10.3389/fmed.2025.1500389 10.3389/fmed.2025.1500389 PMC12163005 40520800

[16] Patrick DL, Burke LB, Gwaltney CJ, Leidy NK, Martin ML, Molsen E et al (2011) Content Validity—Establishing and Reporting the Evidence in Newly Developed Patient-Reported Outcomes (PRO) Instruments for Medical Product Evaluation: ISPOR PRO Good Research Practices Task Force Report: Part 1—Eliciting Concepts for a New PRO Instrument. Value Health 14(8):967–977. 10.1016/j.jval.2011.06.014 22152165 10.1016/j.jval.2011.06.014

[17] U.S. Department of Health and Human Services FDA Center for Drug Evaluation and Research, U.S. Department of Health and Human Services FDA Center for Biologics Evaluation and Research, U.S. Department of Health and Human Services FDA Center for Devices and Radiological Health (2006) Guidance for industry: patient-reported outcome measures: use in medical product development to support labeling claims: draft guidance. Health Qual Life Outcomes 4(1):79. 10.1186/1477-7525-4-79 17034633 10.1186/1477-7525-4-79 PMC1629006

[18] Terwee CB, Prinsen CAC, Chiarotto A, Westerman MJ, Patrick DL, Alonso J et al (2018) COSMIN methodology for evaluating the content validity of patient-reported outcome measures: a Delphi study. Qual Life Res 27(5):1159–1170. 10.1007/s11136-018-1829-0 29550964 10.1007/s11136-018-1829-0 PMC5891557

[19] Palinkas LA, Horwitz SM, Green CA, Wisdom JP, Duan N, Hoagwood K (2015) Purposeful Sampling for Qualitative Data Collection and Analysis in Mixed Method Implementation Research. Adm Policy Ment Health 42(5):533–544. 10.1007/s10488-013-0528-y 24193818 10.1007/s10488-013-0528-y PMC4012002

[20] Guest G, Bunce A, Johnson L (2006) How Many Interviews Are Enough? An Experiment with Data Saturation and Variability. Field Methods 18(1):59–82. 10.1177/1525822X05279903

[21] Kallio H, Pietilä A, Johnson M, Kangasniemi M (2016) Systematic methodological review: developing a framework for a qualitative semi-structured interview guide. J Adv Nurs 72(12):2954–2965. 10.1111/jan.13031 27221824 10.1111/jan.13031

[22] DiCicco-Bloom B, Crabtree BF (2006) The qualitative research interview. Med Educ 40(4):314–321. 10.1111/j.1365-2929.2006.02418.x 16573666 10.1111/j.1365-2929.2006.02418.x

[23] Mayring P (2014) Qualitative content analysis - theoretical foundation, basic procedures and software solution

[24] Rugg G, McGeorge P (2005) The sorting techniques: A tutorial paper on card sorts, picture sorts and item sorts. Expert Syst 22:94–107. 10.1111/1468-0394.00045

[25] Beaton DE, Bombardier C, Guillemin F, Ferraz MB (2000) Guidelines for the process of cross-cultural adaptation of self-report measures. Spine (Phila Pa 1976) 25(24):3186–3191. 10.1097/00007632-200012150-00014 PubMed PMID: 11124735 11124735 10.1097/00007632-200012150-00014

[26] Nunnally JC, Bernstein IH (1994) Psychometric theory. 3. ed., [Nachdr.]. New York, NY: McGraw-Hill; 752 p. (McGraw-Hill series in psychology)

[27] Koo TK, Li MY (2016) A Guideline of Selecting and Reporting Intraclass Correlation Coefficients for Reliability Research. J Chiropr Med 15(2):155–163. 10.1016/j.jcm.2016.02.012 27330520 10.1016/j.jcm.2016.02.012 PMC4913118

[28] Beatty PC, Willis GB (2007) Research Synthesis: The Practice of Cognitive Interviewing. Pub Opin Q 71(2):287–311. 10.1093/poq/nfm006

[29] Willis G (2005) Cognitive Interviewing: A Tool For Improving Questionnaire Design. Sage, Thousand Oaks, Calif.

[30] Stein REK, Riessman CK (1980) The Development of an Impact-on-Family Scale: Preliminary Findings. Med Care 18(4):465–472. 10.1097/00005650-198004000-00010 7401703 10.1097/00005650-198004000-00010

[31] Varni JW, Sherman SA, Burwinkle TM, Dickinson PE, Dixon P (2004) The PedsQL^™^ Family Impact Module: Preliminary reliability and validity. Health Qual Life Outcomes 2(1):55. 10.1186/1477-7525-2-55 15450120 10.1186/1477-7525-2-55 PMC521692

