# Supplementary material for: Development and pilot testing of the quality of life of parents of children with achondroplasia questionnaire
Source: J Patient Rep Outcomes. 2026 Jun 19;10:101. doi: 10.1186/s41687-026-01127-9 (PMC13282410; doi:10.1186/s41687-026-01127-9)
Supplement: Supplementary file 1 — Supplementary Material 1 [file 41687_2026_1127_MOESM1_ESM.docx]

**Supplementary Material: Development and Pilot Testing of the Quality of Life of Parents of Children with Achondroplasia Questionnaire**

**Supplementary Materials**

**Supplementary Table 1.**

*Qualitative–Quantitative Traceability of the QOLA Questionnaire*

| **QOLA Domain (Final)** | **Phase 1 Code** | **Phase 2 Higher-Order Category** | **Illustrative Quote (English)** | **Item ID(s)** | **Final QOLA Item(s)** |
| --- | --- | --- | --- | --- | --- |
| **Healthcare experiences** | Health insurance and authorities | Health care system and bureaucracy | “Dealing with care levels and disability status feels like a constant battle.” | QOLA-H1, QOLA-H8 | I am satisfied with the health care system concerning my child's needs. / Acquiring official recognition for my child's disability is stressful for me. |
|  | Bureaucracy | Health care system and bureaucracy | “Managing paperwork for benefits feels like a part-time office job.” | QOLA-H7 | Managing my child's healthcare requires a significant amount of my time. |
|  | Administrative mismatch | Health care system and bureaucracy | “The forms are designed for elderly people, not for families with young children.” | QOLA-H9 | The process of accessing specialised healthcare for my child’s needs is difficult. |
|  | Diagnostic communication | Diagnostic experience | “The diagnosis was communicated very abruptly, and I felt completely overwhelmed.” | QOLA-H5 | I trust the information provided by medical staff. |
|  | Medical decision-making | Medical decision-making and responsibility | “It was important that the child was involved, because they have to endure the treatment.” | QOLA-H3, QOLA-MH9 | I am confident in the medical treatment my child receives. / I find it challenging to make medical decisions for my child. |
| **Challenges and support** | Purchases | Environmental adaptation | “Adapting the home so my child can be independent takes enormous time and effort.” | QOLA-CS1, QOLA-CS2 | The changes needed in my home to accommodate my child's special needs are overwhelming. / Implementing the necessary changes in my home is difficult. |
|  | School and childcare | Social environment and inclusion | “Despite inclusive policies, everyday school life was often not barrier-free.” | QOLA-CS3 | Coordinating with the school/kindergarten to optimise my child's care is stressful. |
|  | Social environment | Social environment and inclusion | “Despite inclusive policies, everyday school life was often not barrier-free.” | QOLA-CS4 | The public spaces are suitable for my child's special needs. |
|  | Support needs (knowledge) | Support needs and resources | “I wish doctors were better informed about what achondroplasia really means.” | QOLA-CS5 | Information about medical treatments for my child is easy to access. |
|  | Financial strain | Parental burden and coping | “Managing everyday expenses is stressful.” | QOLA-CS6 | I am financially stressed due to the expenses of my child's special needs. |
|  | Purchases | Environmental adaptation | “Finding everyday items is much harder.” | QOLA-CS7 | It's hard to find everyday items like clothing or shoes for my child. |
| **Physical health** | Physical strain | Parental health and well-being | “Carrying my child during treatment phases caused lasting back and shoulder pain.” | QOLA-PH5, QOLA-PH6 | Caring for my child is physically exhausting due to their special needs. / Supporting my child's mobility causes me physical pain. |
|  | Physical strain | Parental health and well-being | “I feel worn out most days.” | QOLA-PH1–PH4 | I am satisfied with my physical health. / physical activity level / sleep quality / energy for daily activities. |
| **Mental health** | Psychological strain | Parental health and well-being | “During intensive treatment phases, I felt emotionally exhausted and depleted.” | QOLA-MH3 | Caring for my child is mentally exhausting due to their special needs. |
|  | Feelings after diagnosis | Emotional processing and adjustment | “At first, there was complete despair.” | QOLA-MH4 | I feel overwhelmed by stress related to my child’s medical procedures. |
|  | Adjustment over time | Emotional processing and adjustment | “Over time, the worries became less overwhelming.” | QOLA-MH1 | I am satisfied with my mental health in relation to my child's achondroplasia. |
|  | Social concerns | Social environment and inclusion | “I worry how others treat my child.” | QOLA-MH6, QOLA-MH7 | I am concerned about my child being treated unfair. / I worry how others react to my child’s appearance. |
|  | Protectiveness | Family dynamics | “I am careful who I leave my child with.” | QOLA-MH8 | I worry about leaving my child with others outside school or kindergarten. |
| **Social life and relationships** | Social reactions and stigma | Social environment and inclusion | “Constantly explaining and advocating is exhausting.” | QOLA-SR1, QOLA-SR2 | I feel exhausted by attention and stares. / I feel tired of explaining achondroplasia. |
|  | Differential treatment | Social environment and inclusion | “People treat us differently.” | QOLA-SR5 | I experience significant differential treatment in my social life. |
|  | Peer and self-help support | Social support and networking | “Meeting other families helped enormously.” | QOLA-SR3, QOLA-SR6 | I am satisfied with the accessibility of support groups. / emotional support from others. |
|  | Everyday life | Everyday organisation and routines | “There’s little time left for ourselves.” | QOLA-SR7, QOLA-SR8 | My leisure opportunities are limited. / My personal relationships are limited. |
| **Coping** | Perceived benefits | Parental burden and coping | “I realised these children are not unhappy.” | QOLA-C1 | I feel that having a child with achondroplasia enriches my life. |
|  | Practical advantages | Parental burden and coping | “Support makes things easier.” | QOLA-C2 | Getting subsidies and tax benefits is helpful. |
|  | Support and adjustment | Support needs and resources | “We learned to manage better.” | QOLA-C3–C6 | I have enough support. / I can manage caregiving time. / My family supports me. / My family is coping well. |
| **Family and daily life** | Family impact | Family dynamics | “Achondroplasia affects the whole family.” | QOLA-FD8, QOLA-FD9 | Having a child has positively affected family relationships. / My family feels stressed. |
|  | Siblings | Family dynamics | “Everything revolves around one child.” | QOLA-FD7 | I am satisfied with relationships with all children in my household. |
|  | Everyday life | Everyday organisation and routines | “Daily life is more stressful.” | QOLA-FD1–FD5, FD10 | My everyday life/work/capacity is affected. / Medical appointments are a burden. / Leisure is restricted. |
| **Worries and future concerns** | General future worries | Future-related concerns | “We worry about bullying and independence.” | QOLA-WF1, WF6, WF7 | I worry about doing enough, independence, negative social experiences. |
|  | Child’s future | Future-related concerns | “Education and work are always in mind.” | QOLA-WF2–WF5, WF8 | I worry about relationships, appointments, exclusion, social benefits. |

**Supplementary Table 2.**

*Initial factor loadings (EFA) of pilot version (63-items) and iterative item reduction process per subscale*

| **Subscale** | **Item** | **Initial factor loading (EFA)** | **Subscale α (if deleted)** | **Step Removed** | **Final Status** |
| --- | --- | --- | --- | --- | --- |
| Healthcare Experiences | HE01 | .886 | .580 | - | Retained |
|  | HE02 | .834 | .582 | - | Retained |
|  | HE03 | .686 | .633 | - | Retained |
|  | HE04 | .294 | .716 | Step 1 | Removed |
|  | HE05 | .830 | .607 | - | Retained |
|  | HE06 | .436 | .792 | Step 2 | Removed |
|  | HE07 | .387 | .790 | Step 2 | Removed |
|  | HE08 | .063 | .741 | Step 1 | Removed |
|  | HE09 | .563 | .859 | Step 3 | Removed |
| Challenges & support | CS01 | .737 | .573 | - | Retained |
|  | CS02 | .614 | .586 | - | Retained |
|  | CS03 | .597 | .608 | - | Retained |
|  | CS04 | .444 | .649 | Step 1 | Removed |
|  | CS05 | .289 | .674 | Step 1 | Removed |
|  | CS06 | .634 | .588 | - | Retained |
|  | CS07 | .624 | .608 | - | Retained |
| Physical health | PH01 | .798 | .504 | - | Retained |
|  | PH02 | .631 | .588 | - | Retained |
|  | PH03 | .277 | .685 | Step 1 | Removed |
|  | PH04 | .638 | .580 | - | Retained |
|  | PH05 | .669 | .591 | - | Retained |
|  | PH06 | .564 | .611 | - | Retained |
| Mental health | MH01 | .652 | .660 | - | Retained |
|  | MH02 | .440 | .721 | Step 2 | Removed |
|  | MH03 | .500 | .682 | - | Retained |
|  | MH04 | .771 | .637 | - | Retained |
|  | MH05 | .337 | .713 | Step 1 | Removed |
|  | MH06 | .624 | .673 | - | Retained |
|  | MH07 | .526 | .693 | - | Retained |
|  | MH08 | .651 | .668 | - | Retained |
|  | MH09 | .428 | .713 | Step 2 | Removed |
| Social interactions | SI00 | .440 | .611 | Step 1 | Removed |
|  | SI01 | .418 | .618 | Step 1 | Removed |
|  | SI02 | .227 | .654 | Step 1 | Removed |
|  | SI03 | .490 | .622 | - | Retained |
|  | SI04 | .644 | .603 | - | Retained |
|  | SI05 | .613 | .578 | - | Retained |
|  | SI06 | .568 | .605 | - | Retained |
|  | SI07 | .763 | .563 | - | Retained |
| Coping | Co01 | -.149 | .715 | Step 1 | Removed |
|  | Co02 | .617 | .538 | - | Retained |
|  | Co04 | .697 | .549 | - | Retained |
|  | Co05 | .532 | .577 | - | Retained |
|  | Co06 | .815 | .507 | - | Retained |
|  | Co07 | .779 | .543 | - | Retained |
| Daily functioning | FD01 | .800 | .786 | - | Retained |
|  | FD02 | .692 | .800 | - | Retained |
|  | FD03 | .797 | .784 | - | Retained |
|  | FD04 | .724 | .792 | - | Retained |
|  | FD05 | .575 | .813 | - | Retained |
|  | FD06 | .597 | .812 | - | Retained |
|  | FD07 | .389 | .833 | Step 2 | Removed |
|  | FD09 | .346 | .825 | Step 1 | Removed |
|  | FD10 | .548 | .809 | Step 3 | Removed |
|  | FD11 | .713 | .795 | - | Retained |
| Future worries | WF01 | .465 | .789 | Step 1 | Removed |
|  | WF02 | .784 | .745 | - | Retained |
|  | WF03 | .708 | .758 | - | Retained |
|  | WF04 | .595 | .776 | - | Retained |
|  | WF05 | .817 | .732 | - | Retained |
|  | WF06 | .702 | .746 | - | Retained |
|  | WF07 | .757 | .757 | - | Retained |
|  | WF08 | .333 | .794 | Step 1 | Removed |

Note. Initial factor loadings are derived from the first exploratory factor analysis prior to item reduction. Items were iteratively removed based on (a) factor loadings < .40, (b) improvement in subscale’s reliability (Cronbach’s α), and (c) conceptual considerations. “Subscale α (if deleted**)”** refers to overall subscale’s Cronbach’s alpha if the item were removed. The item with the weakest psychometric performance was removed at each iterative step, and the model was re-estimated before proceeding to the next step. Step 1: Initial reduction; Step 2: Post re-estimation; Step 3: Final reduction.

**Supplementary Table 3.**

*Internal consistency of subscales pre- and post-item-refinement process*

| **Subscale** | **Initial** α | **Post- Step 1** | **Post- Step 2** | **Post- Step 3** | **Final (field-test version)** |
| --- | --- | --- | --- | --- | --- |
| HE | .671 | .779 | .819 | .859 | .859 |
| CS | .650 | .675 | - | - | .675 |
| PH | .639 | .685 | - | - | .685 |
| MH | .706 | .713 | .721 | - | .721 |
| SI | .640 | .654 | .652 | - | .652 |
| Co | .642 | .715 | - | - | .715 |
| FD | .821 | .825 | .833 | .835 | .835 |
| WF | .786 | .807 | - | - | .807 |

Note. Step 1: Initial reduction; Step 2: Post re-estimation; Step 3: Final reduction

**Supplementary Table 4.**

*Item reduction from pilot version (63 items) to field-test version (43 items)*

| **Domain** | **Item code** | **Item content (short)** | **Decision** | **Reason for decision** |
| --- | --- | --- | --- | --- |
| Healthcare experiences | HE01 | Satisfaction with healthcare system | Retained | Strong factor loading and conceptual fit |
|  | HE02 | Collaboration of medical staff | Retained | Strong factor loading and conceptual fit |
|  | HE03 | Confidence in specialised care | Retained | Strong factor loading and conceptual fit |
|  | HE05 | Trust in medical information | Retained | Improved domain coherence after wording refinement |
|  | HE04* | Psychological support during treatment | Removed | Low factor loading and redundancy with HE02/HE03 |
|  | HE06* | Traveling to specialized appointments | Removed | Low loading, improved domain coherence after removal |
|  | HE07* | Care-related time demands | Removed | Low loading, improved domain coherence after removal |
|  | HE08* | Acquiring official recognition | Removed | Weak loading and contextual variability |
|  | HE09* | Difficulty in specialised care access | Removed | Low loading and redundancy |
| Challenges & support | CS01 | Home adaptations overwhelming | Retained | Strong factor loading |
|  | CS02 | Difficulty implementing home changes | Retained | Strong factor loading |
|  | CS03 | Coordination with school/kindergarten | Retained | Strong factor loading |
|  | CS04* | Public spaces suitable | Removed | Poor alignment with domain and low loading |
|  | CS05* | Information easy to access | Removed | Cross-loading and conceptual overlap |
|  | CS06 | Financial stress | Retained | Strong factor loading |
|  | CS07 | Difficulty finding clothing/shoes | Retained | Strong factor loading |
| Physical health | PH01 | Satisfaction with physical health | Retained | Strong factor loading |
|  | PH02 | Satisfaction with activity level | Retained | Strong factor loading |
|  | PH03* | Sleep quality | Removed | Low loading and redundancy |
|  | PH04 | Low energy | Retained | Strong factor loading |
|  | PH05 | Physical exhaustion | Retained | Strong factor loading |
|  | PH06 | Physical pain | Retained | Strong factor loading |
| Mental health | MH01 | Satisfaction with mental health | Retained | Strong factor loading |
|  | MH02* | Difficulty managing daily activities | Removed | Cross-loading with daily functioning |
|  | MH03 | Mental exhaustion | Retained | Strong conceptual relevancy |
|  | MH04 | Stress from medical procedures | Retained | Strong factor loading |
|  | MH05* | Reassurance by inclusion efforts | Removed | Poor domain alignment |
|  | MH06 | Concern about unfair treatment | Retained | Strong factor loading |
|  | MH07 | Worry about others’ reactions | Retained | Strong factor loading |
|  | MH08 | Worry about leaving child with others | Retained | Strong factor loading |
|  | MH09* | Making medical decision | Removed | Low loading, improved domain coherence after removal |
| Social interactions | SI00* | Attention and stares | Removed | Redundancy with stigma-related items |
|  | SI01* | Explaining achondroplasia | Removed | Cross-loading |
|  | SI02* | Support group accessibility | Removed | Poor alignment with domain and low loading |
|  | SI03 | Response to others’ behaviour | Retained | Strong factor loading |
|  | SI04 | Differential treatment | Retained | Strong conceptual relevancy |
|  | SI05 | Emotional support | Retained | Strong factor loading |
|  | SI06 | Limited leisure activities | Retained | Strong factor loading |
|  | SI07 | Limited personal relationships | Retained | Strong factor loading |
| Coping | Co01* | Subsidies/tax benefits | Removed | Weak loading and contextual variability |
|  | Co02 | Emotional enrichment | Retained | Strong factor loading |
|  | Co03* | External practical support | Removed | Redundancy |
|  | Co04 | Support during medical procedures | Retained | Strong factor loading |
|  | Co05 | Managing caregiving time | Retained | Strong factor loading |
|  | Co06 | Family support | Retained | Strong factor loading |
|  | Co07 | Family coping | Retained | Strong factor loading |
| Daily functioning | FD01 | Negative impact on daily life | Retained | Strong factor loading |
|  | FD02 | Negative impact on work life | Retained | Strong factor loading |
|  | FD03 | Reduced work capacity | Retained | Strong factor loading |
|  | FD04 | Increased daily stress | Retained | Strong factor loading |
|  | FD05 | Medical appointments affect life | Retained | Strong factor loading |
|  | FD06 | Relationship with child | Retained | Conceptually important, acceptable loading |
|  | FD07* | Relationships with all children | Removed | Poor alignment and redundancy |
|  | FD08* | Family relationships positively affected | Removed | Cross-loading |
|  | FD09* | Family stress | Removed | Redundancy with FD04 |
|  | FD10* | Restricted leisure activities (family) | Removed | Overlap with SI06 |
|  | FD11 | Family leisure limitations | Retained | Strong factor loading |
| Future worries | WF01* | Not doing enough | Removed | Cross-loading with coping |
|  | WF02 | Child’s social relationships | Retained | Strong factor loading |
|  | WF03 | Intimate relationships | Retained | Strong factor loading |
|  | WF04 | Missing medical appointments | Retained | Strong factor loading |
|  | WF05 | Exclusion from group activities | Retained | Strong factor loading |
|  | WF06 | Future independence | Retained | Strong factor loading |
|  | WF07 | Negative social experiences | Retained | Strong factor loading |
|  | WF08* | Loss of social benefits | Removed | Context-specific, weak loading |

**Pilot test Correlation Matrices**

**Supplementary Table 5**

*Pilot test correlation matrix – Pooled sample (N = 50)*

|  | | HE | CS | PH | MH | SI | Co | FD | WF |
| --- | --- | --- | --- | --- | --- | --- | --- | --- | --- |
| HE | Pearson Correlation | 1 | -,483^**^ | -,461^**^ | -,537^**^ | -,541^**^ | ,514^**^ | -,513^**^ | -,030 |
|  | Sig. (2-tailed) |  | <,001 | <,001 | <,001 | <,001 | <,001 | <,001 | ,838 |
| CS | Pearson Correlation | -,483^**^ | 1 | ,478^**^ | ,645^**^ | ,462^**^ | -,407^**^ | ,438^**^ | ,437^**^ |
|  | Sig. (2-tailed) | <,001 |  | <,001 | <,001 | <,001 | ,003 | ,001 | ,002 |
| PH | Pearson Correlation | -,461^**^ | ,478^**^ | 1 | ,570^**^ | ,498^**^ | -,383^**^ | ,467^**^ | ,248 |
|  | Sig. (2-tailed) | <,001 | <,001 |  | <,001 | <,001 | ,006 | <,001 | ,082 |
| MH | Pearson Correlation | -,537^**^ | ,645^**^ | ,570^**^ | 1 | ,558^**^ | -,365^**^ | ,490^**^ | ,549^**^ |
|  | Sig. (2-tailed) | <,001 | <,001 | <,001 |  | <,001 | ,009 | <,001 | <,001 |
| SI | Pearson Correlation | -,541^**^ | ,462^**^ | ,498^**^ | ,558^**^ | 1 | -,588^**^ | ,561^**^ | ,245 |
|  | Sig. (2-tailed) | <,001 | <,001 | <,001 | <,001 |  | <,001 | <,001 | ,086 |
| Co | Pearson Correlation | ,514^**^ | -,407^**^ | -,383^**^ | -,365^**^ | -,588^**^ | 1 | -,724^**^ | -,045 |
|  | Sig. (2-tailed) | <,001 | ,003 | ,006 | ,009 | <,001 |  | <,001 | ,756 |
| FD | Pearson Correlation | -,513^**^ | ,438^**^ | ,467^**^ | ,490^**^ | ,561^**^ | -,724^**^ | 1 | ,048 |
|  | Sig. (2-tailed) | <,001 | ,001 | <,001 | <,001 | <,001 | <,001 |  | ,741 |
| WF | Pearson Correlation | -,030 | ,437^**^ | ,248 | ,549^**^ | ,245 | -,045 | ,048 | 1 |
|  | Sig. (2-tailed) | ,838 | ,002 | ,082 | <,001 | ,086 | ,756 | ,741 |  |

**Supplementary Table 6.**

*Pilot test correlation matrix – German (N = 16)*

|  | | HE | CS | PH | MH | SI | Co | FD | WF |
| --- | --- | --- | --- | --- | --- | --- | --- | --- | --- |
| HE | Pearson Correlation | 1 | -,652^**^ | -,618^*^ | -,491 | -,607^*^ | ,557^*^ | -,677^**^ | -,022 |
|  | Sig. (2-tailed) |  | ,006 | ,011 | ,053 | ,013 | ,025 | ,004 | ,935 |
| CS | Pearson Correlation | -,652^**^ | 1 | ,577^*^ | ,550^*^ | ,306 | -,464 | ,311 | ,432 |
|  | Sig. (2-tailed) | ,006 |  | ,019 | ,027 | ,249 | ,070 | ,241 | ,095 |
| PH | Pearson Correlation | -,618^*^ | ,577^*^ | 1 | ,731^**^ | ,706^**^ | -,639^**^ | ,715^**^ | ,264 |
|  | Sig. (2-tailed) | ,011 | ,019 |  | ,001 | ,002 | ,008 | ,002 | ,323 |
| MH | Pearson Correlation | -,491 | ,550^*^ | ,731^**^ | 1 | ,649^**^ | -,695^**^ | ,650^**^ | ,385 |
|  | Sig. (2-tailed) | ,053 | ,027 | ,001 |  | ,006 | ,003 | ,006 | ,141 |
| SI | Pearson Correlation | -,607^*^ | ,306 | ,706^**^ | ,649^**^ | 1 | -,827^**^ | ,815^**^ | ,086 |
|  | Sig. (2-tailed) | ,013 | ,249 | ,002 | ,006 |  | <,001 | <,001 | ,752 |
| Co | Pearson Correlation | ,557^*^ | -,464 | -,639^**^ | -,695^**^ | -,827^**^ | 1 | -,799^**^ | -,404 |
|  | Sig. (2-tailed) | ,025 | ,070 | ,008 | ,003 | <,001 |  | <,001 | ,121 |
| FD | Pearson Correlation | -,677^**^ | ,311 | ,715^**^ | ,650^**^ | ,815^**^ | -,799^**^ | 1 | ,125 |
|  | Sig. (2-tailed) | ,004 | ,241 | ,002 | ,006 | <,001 | <,001 |  | ,644 |
| WF | Pearson Correlation | -,022 | ,432 | ,264 | ,385 | ,086 | -,404 | ,125 | 1 |
|  | Sig. (2-tailed) | ,935 | ,095 | ,323 | ,141 | ,752 | ,121 | ,644 |  |

**Supplementary Table 7.**

*Pilot test correlation matrix – Portuguese (N = 17)*

|  | | HE | CS | PH | MH | SI | Co | FD | WF |
| --- | --- | --- | --- | --- | --- | --- | --- | --- | --- |
| HE | Pearson Correlation | 1 | -,569^*^ | -,330 | -,679^**^ | -,672^**^ | ,567^*^ | -,518^*^ | -,074 |
|  | Sig. (2-tailed) |  | ,017 | ,196 | ,003 | ,003 | ,018 | ,033 | ,779 |
| CS | Pearson Correlation | -,569^*^ | 1 | ,536^*^ | ,738^**^ | ,697^**^ | -,220 | ,449 | ,429 |
|  | Sig. (2-tailed) | ,017 |  | ,027 | <,001 | ,002 | ,396 | ,071 | ,086 |
| PH | Pearson Correlation | -,330 | ,536^*^ | 1 | ,655^**^ | ,571^*^ | -,095 | ,209 | ,460 |
|  | Sig. (2-tailed) | ,196 | ,027 |  | ,004 | ,017 | ,716 | ,421 | ,063 |
| MH | Pearson Correlation | -,679^**^ | ,738^**^ | ,655^**^ | 1 | ,733^**^ | -,384 | ,446 | ,326 |
|  | Sig. (2-tailed) | ,003 | <,001 | ,004 |  | <,001 | ,128 | ,073 | ,201 |
| SI | Pearson Correlation | -,672^**^ | ,697^**^ | ,571^*^ | ,733^**^ | 1 | -,416 | ,444 | ,258 |
|  | Sig. (2-tailed) | ,003 | ,002 | ,017 | <,001 |  | ,097 | ,074 | ,317 |
| Co | Pearson Correlation | ,567^*^ | -,220 | -,095 | -,384 | -,416 | 1 | -,672^**^ | ,167 |
|  | Sig. (2-tailed) | ,018 | ,396 | ,716 | ,128 | ,097 |  | ,003 | ,521 |
| FD | Pearson Correlation | -,518^*^ | ,449 | ,209 | ,446 | ,444 | -,672^**^ | 1 | -,065 |
|  | Sig. (2-tailed) | ,033 | ,071 | ,421 | ,073 | ,074 | ,003 |  | ,805 |
| WF | Pearson Correlation | -,074 | ,429 | ,460 | ,326 | ,258 | ,167 | -,065 | 1 |
|  | Sig. (2-tailed) | ,779 | ,086 | ,063 | ,201 | ,317 | ,521 | ,805 |  |

**Supplementary Table 8.**

*Pilot test correlation matrix – Italian (N = 17)*

|  | | HE | CS | PH | MH | SI | Co | FD | WF |
| --- | --- | --- | --- | --- | --- | --- | --- | --- | --- |
| HE | Pearson Correlation | 1 | -,260 | -,362 | -,529^*^ | -,468 | ,683^**^ | -,421 | -,203 |
|  | Sig. (2-tailed) |  | ,313 | ,154 | ,029 | ,058 | ,002 | ,092 | ,435 |
| CS | Pearson Correlation | -,260 | 1 | ,414 | ,728^**^ | ,451 | -,432 | ,489^*^ | ,702^**^ |
|  | Sig. (2-tailed) | ,313 |  | ,099 | <,001 | ,070 | ,083 | ,046 | ,002 |
| PH | Pearson Correlation | -,362 | ,414 | 1 | ,501^*^ | ,300 | -,570^*^ | ,535^*^ | ,508^*^ |
|  | Sig. (2-tailed) | ,154 | ,099 |  | ,041 | ,242 | ,017 | ,027 | ,037 |
| MH | Pearson Correlation | -,529^*^ | ,728^**^ | ,501^*^ | 1 | ,600^*^ | -,523^*^ | ,757^**^ | ,811^**^ |
|  | Sig. (2-tailed) | ,029 | <,001 | ,041 |  | ,011 | ,031 | <,001 | <,001 |
| SI | Pearson Correlation | -,468 | ,451 | ,300 | ,600^*^ | 1 | -,340 | ,247 | ,666^**^ |
|  | Sig. (2-tailed) | ,058 | ,070 | ,242 | ,011 |  | ,182 | ,340 | ,004 |
| Co | Pearson Correlation | ,683^**^ | -,432 | -,570^*^ | -,523^*^ | -,340 | 1 | -,430 | -,428 |
|  | Sig. (2-tailed) | ,002 | ,083 | ,017 | ,031 | ,182 |  | ,085 | ,086 |
| FD | Pearson Correlation | -,421 | ,489^*^ | ,535^*^ | ,757^**^ | ,247 | -,430 | 1 | ,498^*^ |
|  | Sig. (2-tailed) | ,092 | ,046 | ,027 | <,001 | ,340 | ,085 |  | ,042 |
| WF | Pearson Correlation | -,203 | ,702^**^ | ,508^*^ | ,811^**^ | ,666^**^ | -,428 | ,498^*^ | 1 |
|  | Sig. (2-tailed) | ,435 | ,002 | ,037 | <,001 | ,004 | ,086 | ,042 |  |
